# Protein chainmail variants in dsDNA viruses

**DOI:** 10.3934/biophy.2015.2.200

**Published:** 2015-06-17

**Authors:** Z. Hong Zhou, Joshua Chiou

**Affiliations:** 1 Department of Microbiology, Immunology and Molecular Genetics, University of California, Los Angeles, California 90095, USA; 2 California NanoSystems Institute (CNSI), University of California, Los Angeles (UCLA), Los Angeles, CA, 90095, USA

**Keywords:** structural biology, microbiology, protein chainmail, HK97, BPP-1, P22, lambda, Herpesvirus, RRV, HK97-like fold, virus, cryoEM, X-ray crystallography

## Abstract

First discovered in bacteriophage HK97, biological chainmail is a highly stable system formed by concatenated protein rings. Each subunit of the ring contains the HK97-like fold, which is characterized by its submarine-like shape with a 5-stranded β sheet in the axial (A) domain, spine helix in the peripheral (P) domain, and an extended (E) loop. HK97 capsid consists of covalently-linked copies of just one HK97-like fold protein and represents the most effective strategy to form highly stable chainmail needed for dsDNA genome encapsidation. Recently, near-atomic resolution structures enabled by cryo electron microscopy (cryoEM) have revealed a range of other, more complex variants of this strategy for constructing dsDNA viruses. The first strategy, exemplified by P22-like phages, is the attachment of an insertional (I) domain to the core 5-stranded β sheet of the HK97-like fold. The atomic models of the Bordetella phage BPP-1 showcases an alternative topology of the classic HK97 topology of the HK97-like fold, as well as the second strategy for constructing stable capsids, where an auxiliary jellyroll protein dimer serves to cement the non-covalent chainmail formed by capsid protein subunits. The third strategy, found in lambda-like phages, uses auxiliary protein trimers to stabilize the underlying non-covalent chainmail near the 3-fold axis. Herpesviruses represent highly complex viruses that use a combination of these strategies, resulting in four-level hierarchical organization including a non-covalent chainmail formed by the HK97-like fold domain found in the floor region. A thorough understanding of these structures should help unlock the enigma of the emergence and evolution of dsDNA viruses and inform bioengineering efforts based on these viruses.

## 1. Protein Chainmail and the HK97-like Fold

**Figure 1:**
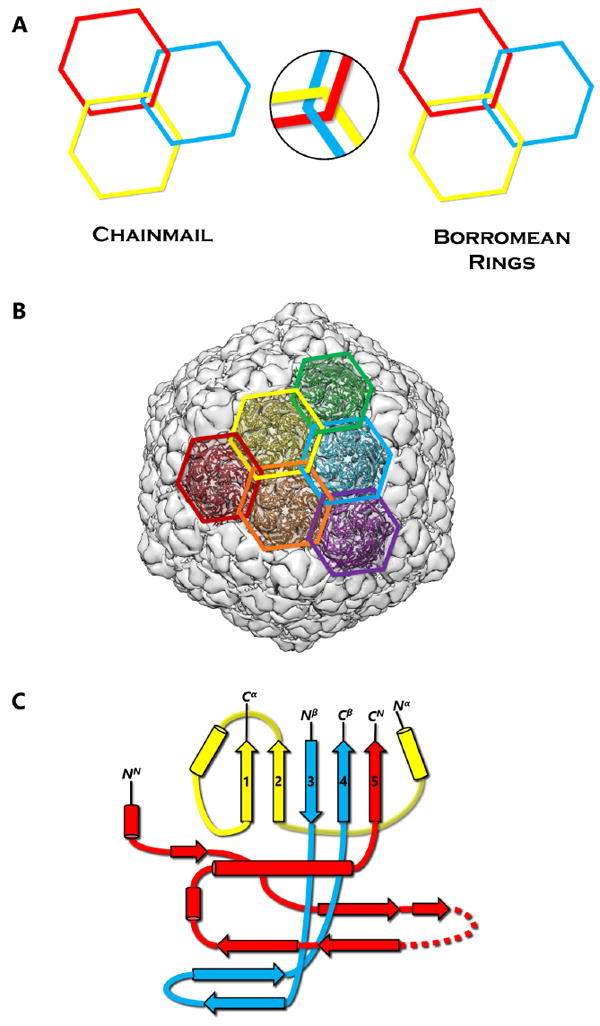
Protein Chainmail and the HK97-like fold. (A) Concept of chainmail versus Borromean rings. Breaking one ring in a chainmail would not affect the integrity of the whole, unlike the case of Borromean rings. (B) Protein chainmail of HK97. Chainmail is a structural organization of concatenated rings found in the capsids of icosahedral dsDNA viruses, and allow capsids to withstand internal forces exerted by the dsDNA. The interlocking rings of protein chainmail resemble armor constructed of metal rings worn by medieval knights during times of battle. (C) Basic building blocks of the HK97-like fold include the N (red), α (yellow), and β (blue) primary elements. Variations of the HK97-like fold can occur when the basic building blocks are connected in different order. Additionally, extra domains can be inserted at the E-loop or at the ends of the building blocks.

Chainmail is a system formed by concatenated rings (Figure 1A). Chainmail in the form of interlocking rings of metal was used by medieval knights to protect their bodies from external forces in battle. The term protein chainmail was coined to explain how capsid proteins of HK97 form large complexes that behave abnormally under biochemical analyses [1]. Later, the X-ray structure revealed how the polypeptide chains are arranged in the HK97 mature capsid to form concatenated rings [2] (Figure 1B). Since then, protein chainmail has been found in the capsids of other icosahedral double-stranded DNA (dsDNA) viruses and protein cages [3,4]. Like the armor, chainmail in viruses provides a thin yet durable layer to endure the internal force exerted by the encapsulated dsDNA [5]. In both cases, chainmail may have been developed for its ability to maintain structural integrity. Indeed, unlike Borromean rings, breaking a single ring in a chainmail does not affect the integrity of the whole mail (Figure 1A).

Each protein ring is composed of five or six subunits (pentamers and hexamers respectively) (Figure 1B), linked by either covalent or non-covalent interactions. First discovered in bacteriophage HK97 in 2000 [2], the HK97-like fold of the subunit has the shape of a modern submarine with a turret, a large forward and a small tail section (Figure 1C). The turret, forward, and tail correspond to the axial (A) domain, peripheral (P) domain and extended (E) loop, respectively. Prominent secondary structure features include a spine helix and a 3-stranded β sheet in the P-domain, a 5-stranded β sheet in the A-domain, the hairpin shaped E-loop and an N-terminal extension (N-arm) parallel to the spine helix (Figure 1C).

The HK97-like fold can also be divided into three building blocks—the N, α, and β primary elements (shown in red, yellow, and blue respectively in Figure 1C) to facilitate comparisons of subunits from different viruses [6]. Because the N terminus (and therefore the N building block) is fixed, the ends of 4 β strands and one α helix in the A-domain can only join in two distinct ways, resulting in two different topologies of the HK97-like fold [6]. The HK97-like fold of the HK97 subunit is connected in the order N, α, and β. This contrasts with the HK97-like fold in BPP-1, which is arranged in the order N, β and α, representing a non-circular permutation of the order in HK97 [6]. In other words, the topology of the HK97-like fold can only be interchanged between α and β building blocks.

Chainmail has been classified into two different subcategories on the basis of chemical interactions of its subunits: covalent and non-covalent chainmail [7]. Since its discovery in 2000 [2], the presence of protein chainmail in dsDNA phages has been observed in many dsDNA viruses, such as HK97 [2], BPP-1 [6], phage λ [8], P22 [9], and herpesviruses [7,10]. Each of these viruses employs a protein chainmail that may vary in the number of rings but shares a common HK97-like capsid protein fold. As illustrated in Figure 1C, alternating the protein chain connectivity and inserting additional domains to the core HK97-like fold can produce different viral and cellular proteins. This review aims to examine the conservation of the HK97-like fold in dsDNA viruses and cellular proteins and the divergence in the organization of this fold for chainmail construction. Careful examination of existing high-resolution structures of dsDNA viruses reveal the following four different strategies to build protein chainmail with the HK97-like fold.

## 2. Covalent Chainmail in HK97 Formed by Isopeptide Bonds

**Figure 2:**
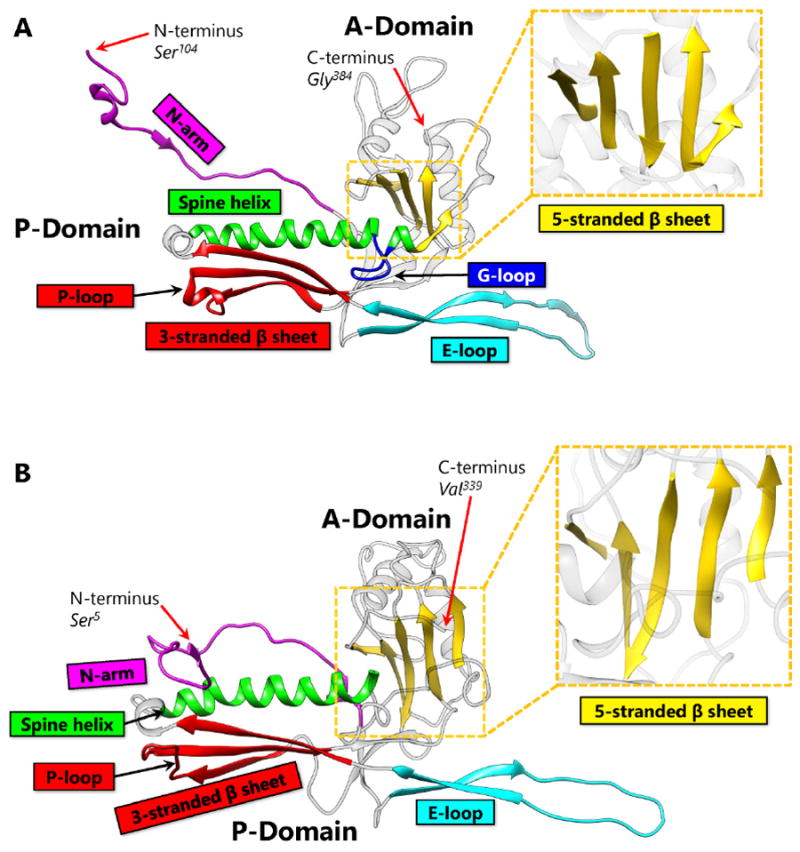
Capsid Protein Comparison Between HK97 and BPP-1. (A) The capsid protein of HK97, gp5 contains the HK97-like fold (Protein Data Bank [PDB] 2FT1). The capsid protein of HK97 is truncated during maturation and begins at Ser_104_, ending at Gly_384_. Major structural elements include the N terminal extension (N-arm, magenta), extended loop (E-loop, cyan), 3-stranded β sheet including the P-loop (red), spine α helix (Spine helix, green), glycine-rich loop (G-loop, blue), and 5-stranded β sheet (zoomed, yellow). Along with the 3-stranded β sheet, the spine helix with a G-loop interruption forms the P-domain. The 5-stranded β sheet forms the core of the A-domain and contains a ↑↑↓↑↑ sheet arrangement. The N-arm and the E-loop protrude from the core of the fold and are flexible to accommodate capsid curvature. (B) The capsid protein of BPP-1 (PDB 3J4U). Unlike gp5 of HK97, the capsid protein of BPP-1 is not truncated. It has the same color scheme as Figure 2A, and contains similar secondary structure elements. The N-arm of BPP-1 is curved such that the β strand can interact with the auxiliary protein (see Figure 5B).

HK97 (named after its place of isolation, Hong Kong) is a long-tailed phage that infects *Escherichia coli* and is a member of the *Siphoviridae* family. The 18-Å thick capsid head is icosahedral with a triangulation number of T = 7 and is composed of 420 capsid protein subunits, forming 12 pentameric and 60 hexameric rings. Historically, protein chainmail for capsid stabilization was originally discovered in HK97, first predicted based on the fact that capsid form covalent complexes and did not enter the gel after denaturation [1] and later visualized at atomic detail by X-ray crystallography [2]. Figure 2A illustrates the typical secondary structural features of the HK97-like fold in the HK97 capsid protein (gp5), while Figure 4A shows a simplified representation showing topology of HK97 HK97-like fold. In addition to these canonical features described above, HK97 gp5 has two other features of note. First, around 100 residues of gp5 are cleaved during maturation [2], leading to a truncated capsid protein. Second, a 10-residue long glycine-rich loop (G-loop) interrupts the tail end of the spine helix. This G-loop has been implicated in the control and guidance of capsid assembly, and has been speculated to be a structural feature sometimes found in viruses with this fold [11].

**Figure 3:**
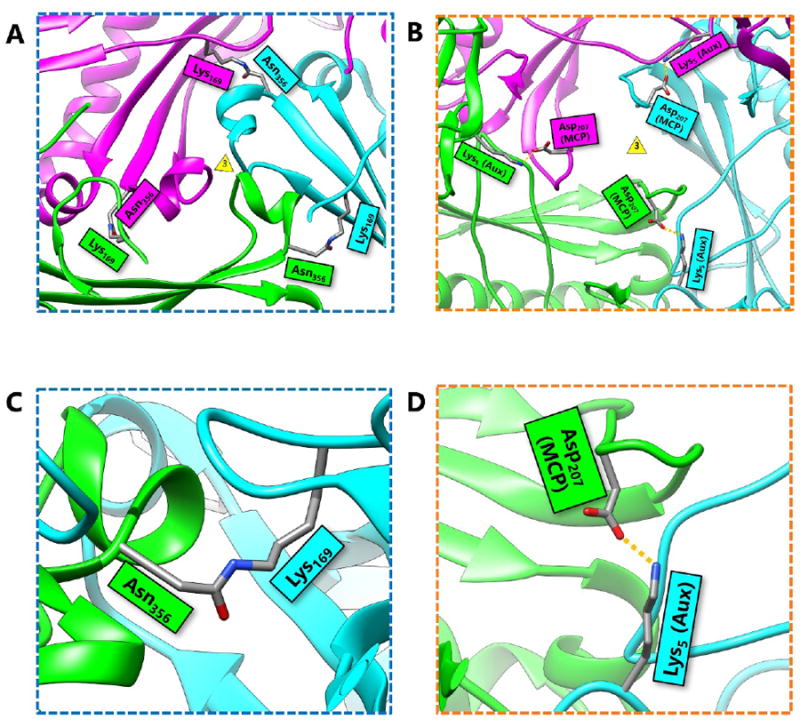
Comparison of HK97 Isopeptide Bonds and BPP-1 Salt Bridges at the 3-fold Axis. (A) A local three-fold interface of HK97 showing the location of isopeptide bonds. The three-fold vertex is indicated by the yellow triangle. Three isopeptide bonds crosslink hexamers and pentamers of the HK97 capsid. Additionally, the protein rings crisscross to form a network of covalently linked, concatenated protein rings, or a covalent chainmail. (B) A local three-fold interface of BPP-1 showing that BPP-1 has salt bridges, but no isopeptide bonds. The salt bridges form between charged residues of the MCP and the auxiliary protein (Aux). These salt bridges, combined with additional non-covalent interactions, complete the non-covalent chainmail of BPP-1. (C) Zoomed-in view of the HK97 isopeptide bond that forms between Lys_169_ and Asn_356_. It functions to chemically bind subunits of HK97 together without the use of additional proteins. (D) Zoomed-in view of the salt bridge in BPP-1 at the three-fold interface. It forms between the positively-charged residue Lys_5_ of the auxiliary protein and the negatively-charged residue Asp_207_ of the capsid protein.

The atomic structure of HK97 capsid revealed that it is stabilized by a combination of van der Waals contacts, hydrogen bonds, salt bridges, and the unique isopeptide bonds, which is a covalent linkage that forms between two amino acid sidechains. In this case, the bond forms between the amino group of K_169_ in the E-loop of one subunit and the carboxyl terminal of N_356_ in the P-loop of another subunit [2]. Near the locations where three hexamers meet (the local/pseudo and global/icosahedral three fold axes, indicated by a triangle in Figure 3), the isopeptide bonds function to chemically bind subunits and anchor the hexamers in place (Figure 3A). In the absence of isopeptide bonds, it has been speculated that the three-fold region represents the weakest point in icosahedral capsids [12]. Thus, it is not surprising that isopeptide bonds bring stability to the capsid at this particular site. Another apparent consequence of these bonds is the overlapping or crisscross of hexamers at the three fold interface. The conjoined E-loops and P-loops coalesce to arrange a system of concatenating rings. A closer inspection of a single isopeptide bond reveals that the subunits are topologically linked [2] (Figure 3C). In the grand scheme of things, all of the subunits are covalently bonded together to form a single molecule—the HK97 capsid. The capsid itself is a network of concatenated, covalently-linked protein rings—a covalent chainmail.

Covalent bonds are resilient relative to their non-covalent counterparts, such as salt bridges or hydrogen bonds, which allows the HK97 capsid to be stable without contracting the use of any auxiliary proteins. This explains how HK97 can utilize a single type of subunit to construct a stable capsid. The strategy that HK97 employs to secure its capsid is both simple and efficient, and thus far, unique among dsDNA virus structures. Given the efficiency of this strategy, and with fast emergence of high-resolution cryoEM structures of dsDNA viruses enabled by direct electron detection technology, it will be just a matter of time before the discovery of similar arrangements of covalent chainmail established by isopeptide bonds in other dsDNA viruses. The availability of such structures in the future should help elucidate the evolutionary origins of the isopeptide bond in viral capsids.

## 3. Non-Covalent Chainmail in P22-like Phages Stabilized by an Insertion

**Figure 4:**
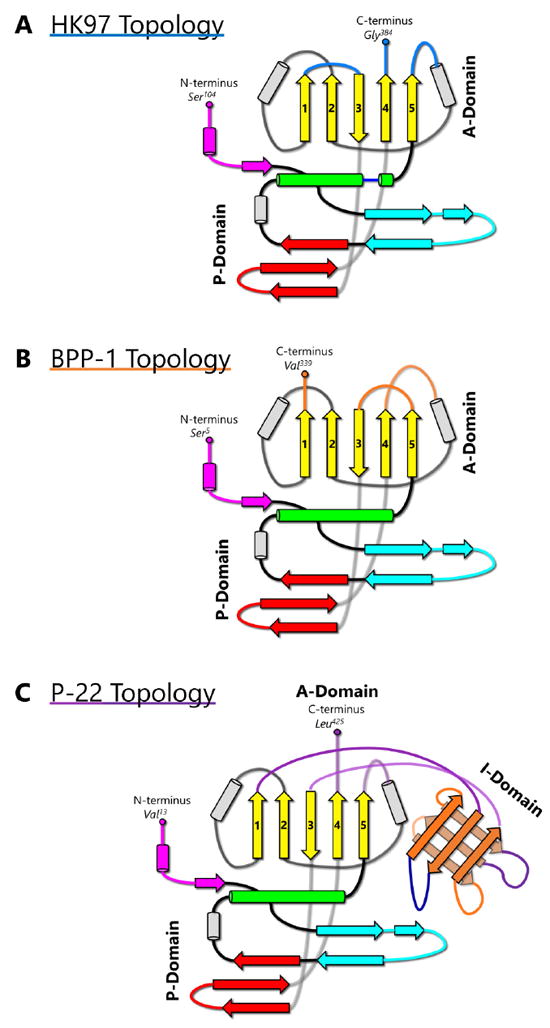
Topology of the HK97-like fold in HK97, BPP-1, and P22. (A) Simplified representation of the topology of the HK97-like fold in HK97. Secondary structural elements are colored according to Fig 2A. (B) Topology of the HK97-like fold in BPP-1 MCP. (C) Topology of the HK97-like fold in P22 capsid protein. The I-domain has insertional junctions at the 5-stranded β sheet after β1 and before β3. The rest of the P22 capsid protein resembles the variant of HK97-like fold found in HK97.

**Figure 5:**
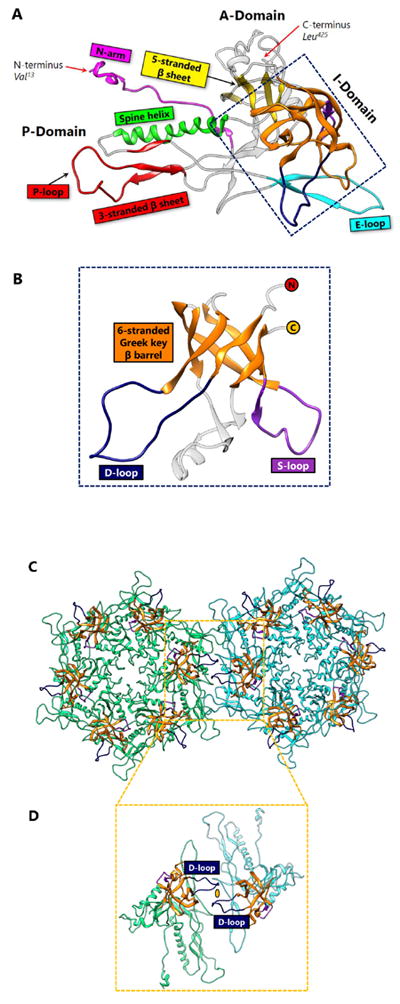
I-domain in P22 Stabilization through Polar Interactions. (A) The HK97-like fold in the capsid protein of P22 includes similar secondary structural elements as HK97 and BPP-1, but also includes an insertional domain (I-domain) with junctions at β1 and β3 of the 5-stranded β sheet. The spine α helix lacks the distinctive G-loop interruption. Instead, the D-loop of the I-domain may replace the function of the G-loop. Additionally, the P-loop appears to be larger than that of HK97 or BPP-1. (B) A view of the I-domain (PDB 2M5S) from the front. The N-terminus (red circle) continues after β1, and the C-terminus (yellow circle) of the I-domain continues to β3. A 6-stranded Greek key β barrel (orange) forms the core of this domain, supported by a D-loop (dark blue) and an S-loop (purple). (C) Twofold interface of the P22 capsid protein. Hexamer 1 (green) and hexamer 2 are held together by polar interactions between the two I-domain D-loops situated across the two-fold axis. The coloring format of the I-domain is the same as Figure 7B. (D) Zoomed-in view removing the other subunits and only showing the two subunits that interact across the two-fold axis. The yellow oval represents the polar interaction that holds the D-loops together.

*Enterobacteriophage* P22 is a *Podoviridae* phage that infects *Salmonella typhimurium*. P22 has been used in molecular biology to study interactions with the host bacterium. Although the P22 capsid protein also contains the conserved HK97-like fold with a topology similar to that of HK97 (Figure 4C), it has a non-conserved accessory insertional domain (I-domain) introduced between the A and P-domains [9] (Figure 5A). The insertional junctions of the I-domain are located at the strands of the five-stranded β sheet of the HK97-like fold core. Interestingly, the insertional domain of P22 is situated at a similar position as the insertion of the additional upper domains in the herpesviruses RRV major capsid protein [7] (see below). The common insertional site within the HK97-like folds of P22 and the herpesviruses suggests a point of modification that may be conserved. This may have significant implications for structural modifications, as insertions can be engineered and attached at this specific position in the HK97-like fold.

The I-domain consists of three main structural elements – an antiparallel six-stranded Greek key β-barrel, the D-loop, and the S-loop (Figure 5B). Although the role of the I-domain has not been firmly established, functional analyses suggest that it is important in capsid protein folding stability, capsid assembly, and capsid size determination [13]. In addition, this domain has been proposed to stabilize individual capsid protein subunits through complementary electrostatic interactions [9], or even to stabilize interactions between adjacent subunits [14]. Positively charged residues of the I-domain, primarily located in the β-barrel, play a role in electrostatic interactions that appear to dock the I-domain to the HK97-like fold core. The D-loop and the S-loop are disordered in the isolated state of the I-domain, but become fixed upon assembly of the virion. It has been suggested that the D-loop plays an analogous role to the G-loop in HK97, and limits conformational changes in the subunit during assembly [13]. The S-loop is somewhat shorter than the D-loop, and plays a role in capsid size determination [13].

At the two-fold interface of the mature capsid (Figure 5C), the I-domain of two P22 subunits forms a dimer complex which are held in place by the polar interactions between the D-loops [13] (Figure 5D). Based on this observation, as well as the fact that the I-domain is rich in β-strands, we hypothesize that the I-domain of P22 is a predecessor of the auxiliary protein in BPP-1 and ε15 (see below). Perhaps the I-domain was cleaved and the product evolved to fasten onto the outer surface of the viral capsid. Conversely, the I-domain could potentially be a derivative of the auxiliary protein, in which case the auxiliary would be presumed to have evolved functionally before the addition of the I-domain. Another plausible explanation is that the I-domain and the auxiliary protein evolved analogously to perform a similar function—to stabilize the subunits through non-covalent interactions. Because the I-domain is observed in P22-like phages, including Sf6 [15] and CUS-3 [16], it has been proposed that the capsid proteins of this phage group have diverged from the classic HK97-like fold [16]. In any case, the P22 capsid provides an example of how the chainmail theme in dsDNA viruses is still maintained despite modifications and augmentations to the basic elementary subunit.

The recently solved structure of the P7 phage lends additional insight into the insertion strategy. Among phages of the P22-like group the location of the insertion is a recurring theme, which P7 also follows. However, instead of having an I-domain insertion, P7 has an A-loop at the 5-stranded β sheet [17]. The A-loop is situated such that a salt bridge can be formed between R_262_ in the A-loop and D_103_ in the 3-stranded β sheet. This intracapsomeric interaction results in non-covalent topological linking [17].

## 4. Non-Covalent Chainmail in BPP-1-like Phages Stabilized by an Auxiliary Protein Dimer

BPP-1 is a short-tailed phage of the *Podoviridae* family and infects *Bordetella pertussis*, the organism responsible for whooping cough. Its capsid is also icosahedral with a T = 7 triangulation number and is comparable in size to that of HK97. However, the atomic structure of BPP-1 reveals two major variations from the structure of the major capsid protein (MCP) of BPP-1 (Figure 2B). The first point of variation is that BPP-1 MCP is not truncated [6], unlike the N-terminal truncation of HK97 capsid protein. Another point of variation lies in the rearranged topology of the HK97-like fold in BPP-1 MCP (Figure 4B). Although the BPP-1 MCP contains the conserved secondary structure elements of the HK97-like fold, it does not contain the same type of G-loop found in HK97. One plausible explanation is that BPP-1 undergoes a different maturation process using a separate scaffolding protein that does not utilize the G-loop.

BPP-1 is not stabilized by covalent bonds, and this difference is highlighted by the lack of isopeptide bonds linking the protein rings. Instead, the capsid of BPP-1 is held together by a network of salt bridges. Salt bridges are intrinsically weaker than covalent bonds in terms of bond dissociation energy. It follows that because salt bridges are energetically weaker than covalent bonds, a greater number of them are required to sustain capsid stability in BPP-1. These salt bridges are found at a similar location at the three-fold interface as the isopeptide bonds in HK97 (Figure 3B). Additionally, salt bridges are found at the two-fold interface, which are supported by the presence of additional proteins.

**Figure 6:**
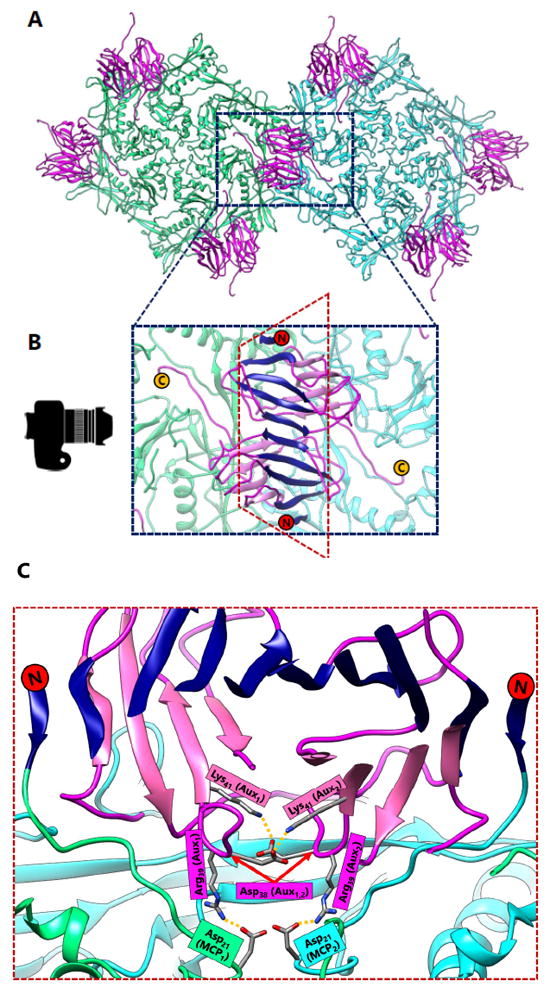
Auxiliary Protein Dimer Interactions in BPP-1. (A) The local two-fold axis of BPP-1. Hexamer 1 (green) and hexamer 2 (cyan) are composed of MCP, and are stabilized by auxiliary proteins (magenta). The auxiliary proteins form dimers across the two-fold axes. (B) Zoomed-in view of the auxiliary protein dimer, which sits atop of MCP subunits at surface of the capsid. The top β strands of the CP dimer form an 8-stranded β sheet. On either side of this 8-stranded β sheet, β strands from the N-terminus (red circle) of capsid proteins hydrogen bond to form an augmented 10-stranded β sheet (navy blue). The C-terminus of the auxiliary protein (yellow circle) is exposed on the outer surface of the capsid, which is beneficial for phage display. (C) When looking from the perspective of the camera and cutting a section of the dimer at the two-fold axis, the interactions between auxiliary and capsid protein become more apparent. A pair of salt bridges hold the auxiliary protein dimer together (Lys_41_–Asp_38_) and salt bridges also form between Asp_21_ of the MCP and Arg_39_ of the auxiliary proteins. In this sense, the auxiliary protein acts like a glue to stabilize the capsid.

The way that the MCP is folded presents a challenge because it limits the orientation and contact of charged residues required to form salt bridges. Thus, the number of salt bridges that can be formed is limited to a finite quantity if the capsid is constructed of only one type of protein. The solution of this problem in BPP-1 is to incorporate an additional protein on the outer surface of the capsid, which is found in a 1 to 1 ratio with the MCP (Figure 6A). This additional protein has a structure that is β sheet rich and contains the jellyroll fold [6]. In previous literature, proteins with similar function have been termed auxiliary [8], decoration [18], stabilizing [19], stapling [20], and cement [6] based on their function. Here we refer to these additional surface proteins as auxiliary proteins to highlight their role in aiding capsid stabilization. These auxiliary proteins form dimers at the local two-fold interface which are held together by hydrogen bonding between the β strands on the edges of the jellyroll β sheets of the two auxiliary proteins (Figure 6B) and a symmetric pair of salt bridges (Figure 6C). The hydrogen bonded N-terminal loops of MCPs on opposing sides of the dimer stabilize this 8-stranded β sheet, forming an augmented 10-stranded β sheet [6]. This augmented β sheet serves as an additional layer of support for the capsid.

More importantly, auxiliary proteins contain charged residues that allow the formation of essential salt bridges with the MCP. At the three-fold interface, Aspartate_207_, which is located in the P-loop of BPP-1 MCP, forms a salt bridge with Lysine_5_, which is found near the N-terminus of the auxiliary protein (Figure 3D). Here, the salt bridge replaces the isopeptide bond of HK97 that binds the P-loop to the E-loop. However, the three-fold non-covalent interactions alone may not be adequate to support the capsid, which may imply the need for additional salt bridges at the two-fold interface (Figure 6C). In this sense, the auxiliary protein may serve as the glue that secures the individual MCPs in place to form the BPP-1 capsid.

The use of additional auxiliary protein to bolster the capsid seems to be a recurring strategy to compensate for the lack of isopeptide bonds in some dsDNA viruses. ε15, which is another phage of similar architecture, also demonstrates a comparable use of auxiliary proteins and salt bridges to support its capsid [20]. Although BPP-1 and ε15 both display the rearranged HK97-like fold, it is unclear whether the auxiliary protein dimer is a necessity of the rearrangement. Protein engineering efforts to revert the BPP-1 topology back to the HK97 topology have not produced functional infectious phage particles [6]. The use of charged residues and hydrogen bonds in the formation of non-covalent chainmail yields functional phages, but such a strategy is nowhere as efficient or as elegant as the covalent bond demonstrated in HK97. Thus, one may consider that such a strategy represents a less-evolved, perhaps more ancestral solution to build protein chainmail.

## 5. Non-Covalent Chainmail in λ-like Phages Stabilized by an Auxiliary Protein Trimer

**Figure 7:**
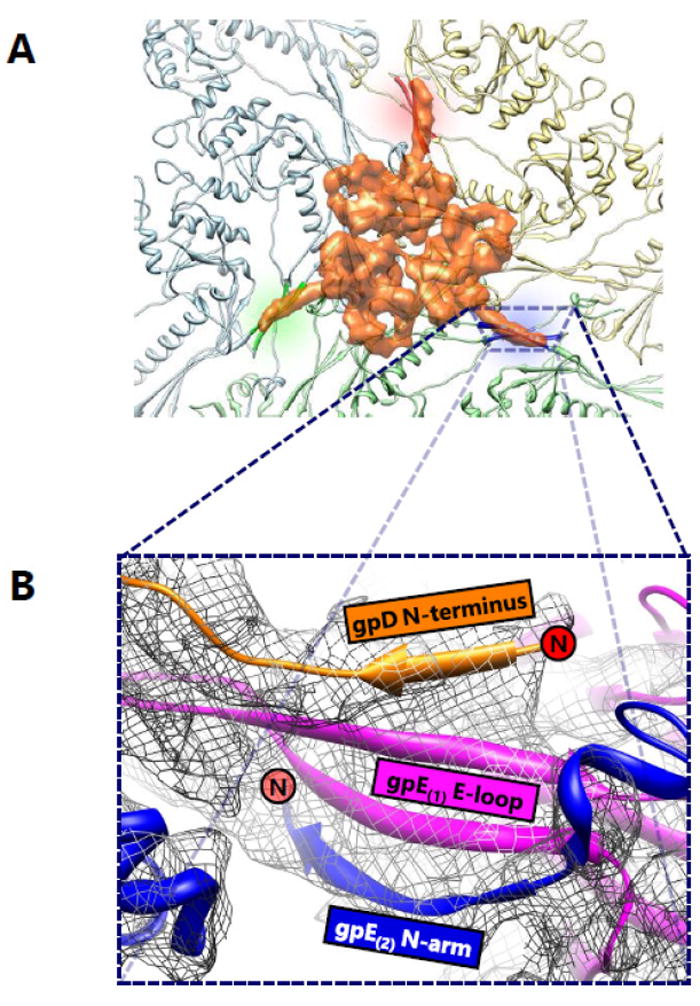
Auxiliary Protein Trimer Interactions in Phage Lambda. (A) A view of the three-fold interface of phage λ from the top. The gpD trimer (orange) is situated atop the site where subunits from three hexamers composed of gpE capsid proteins meet. The gpD trimer interacts with two subunits from each hexamer. In this sense, the gpD trimer acts as a molecular staple and anchors six gpE subunits together. (B) A zoomed-in view of the stabilizing interaction of a single gpD monomer. A four-stranded β sheet is formed from a strand from the gpD N-terminus (orange), two strands from the E-loop of a gpE subunit (magenta), and another strand from the N-arm of an adjacent gpE subunit (blue). The N-termini of gpD and the N-arm of gpE are denoted by red circles. Adapted from [8] with permission from the publisher.

Phage λ is a long-tailed phage with similar morphological characteristics as HK97. It is also a long-tailed *Siphoviridae* phage that infects *E. coli*, and has been well-documented as a model organism for phage-host interactions. The phage λ capsid is constructed of two proteins—gpD, an auxiliary protein and gpE, the MCP. It is not surprising that gpE also displays the HK97-like fold containing secondary structure similarities consistent with other dsDNA phages. Nevertheless, gpE by itself is unable to achieve the chainmail scheme and requires help in the form of an auxiliary protein, gpD. Like BPP-1, phage λ lacks the distinctive chemical linkages of HK97 and instead employs the use of its auxiliary protein gpD. However, a notable difference is that this protein is located at the three-fold rather than the two-fold vertices. The auxiliary protein gpD binds phage λ directly above the three-fold vertices, forming a trimer and stabilizing the mature capsid (Figure 7A). Logically, it would make sense for an auxiliary protein to bind to the three-fold vertex and stabilize the weakest point in icosahedral capsids [12], which is similar in location to isopeptide bonds in HK97 and the salt bridges in BPP-1. In fact, superimposition of the gpD trimer in phage λ with the covalent crosslinks in HK97 revealed that the trimers were positioned directly above the isopeptide bonds [8].

Previously, the gpD monomer was crystallized and its structure was solved at 1.1 Å resolution [19]; however, the crystallized structure was unable to resolve the first fourteen residues of gpD at the N-terminus. These residues, which are critical to the understanding the interaction between gpD and gpE, were shown to be flexible from protein disorder predictions and NMR data [21]. A later cryoEM study resolved the missing fourteen residues and the mystery behind the gpD-gpE interactions [8]. The N-terminus of gpD interacts with gpE by creating a four-stranded β sheet in conjunction with the two β strands of the E-loop from a gpE subunit and the N-terminus β strand from an adjacent gpE subunit (Figure 7B). The contribution of this additional strand from the auxiliary protein gpD functions to staple two gpE subunits together through an augmented β sheet interaction. By taking into account the fact that gpD forms trimeric complexes, it is evident that six subunits from three different gpE rings are secured by non-covalent interactions. As an aside, the limited resolution of the structure presented in [8] does not allow visualization of distinct residues that are involved in these interactions.

The trimeric complex of gpD functions as a molecular staple that binds gpE subunits together to form an augmented β sheet, serving as the basis of the non-covalent chainmail network in phage λ. The auxiliary proteins of both BPP-1 and phage λ utilize the N-terminus of nearby capsid protein subunits to form augmented β sheets, yet the auxiliary protein in BPP-1 forms dimers, while the corresponding auxiliary protein in phage λ forms trimers. To add to the complexity of the situation, the auxiliary proteins in BPP-1 and phage λ are located at the two-fold and three-fold vertices respectively. On the basis of a common stabilization locale at the three-fold vertices, it has been proposed that ancestor of phage λ and HK97 included an auxiliary protein, and that HK97 diverged and evolved covalent crosslinks thereby eliminating the need for an additional protein [8]. In light of the recent discovery of different auxiliary proteins at the two-fold vertices in BPP-1 and ε15, we think that the evolution of chainmail-forming capsids cannot be explained by a simple case of convergent or divergent evolution, but rather a convoluted combination of both.

## 6. A Common Theme and a Possible Strategy to Build Complex Chainmail

All of the viruses utilizing the chainmail strategy contain the conserved HK97-like fold, and can be further categorized, based on the above discussed strategies, by how chainmail is achieved. Presently, HK97 is the only known virus to display covalent chainmail formed with isopeptide crosslinks and thus constitutes its own group (Table 1). The use of a single protein in HK97 to form a stable structure represents a highly optimized strategy, perhaps resulting from evolutionary selection. As such, it may represent a more recent emergence of complexes with the HK97-like fold. Insertional domains, which were originally considered immunoglobulin-like domains in many viruses, are a feature common to the P22-like group of viruses. The BPP-1-like group is classified based on rearrangement of the HK97-like fold and stabilization by means of auxiliary protein dimers at the two-fold interface. Viruses displaying auxiliary protein trimers at the three-fold interface fall under the lambda-like category. More complex viruses such as the herpesviruses utilize a combination of auxiliary proteins, insertional domains, and unique stabilization elements to fasten their capsids.

It appears that viruses utilizing the insertional strategy for capsid stabilization display the widest range of diversity. In contrast to Sf6 [15] and CUS-3 [16], which are similar in capsid size and architecture to P22, the phages T4 [18] and ϕ29 [22] serve as reminders to the diversity witnessed among phages. The prolate capsids of ϕ29 and T4 contain elongated midsections in stark contrast to the hexagonally-shaped capsids of T = 7 phages.

The crystal structure of the capsid protein of the well-studied T4 phage revealed the conserved HK97-like fold with an HK97-like topology [18]. Interestingly, the I-domain in T4 appears to be an extension of the E-loop instead of the 5-stranded β sheet in the A-domain. However, the linker region between the E-loop and I-domain is disordered in the crystal form. Because of the extended formation of the I-domain, it has been hypothesized to make electrostatic contacts with an adjacent HK97-like fold in the same hexamer (or pentamer) based on the complementary nature of both surfaces [18]. If the I-domain in T4 is indeed linked to the E-loop, T4 capsid protein demonstrates another point of modification within the HK97-like fold core. Although the phage ϕ29 is much smaller and less complex than T4, it too displays a prolate capsid composed of capsid proteins containing I-domain [22]. The cryoEM density provides evidence of HK97-like fold secondary structural features, and an additional density deemed a Bacterial Immunoglobulin 2 (BIG2)-like domain. The structural similarity to Ig-like domains is analogous to the case of P22, where the I-domain was originally named a telokin Ig-like domain [9]. The I-domain in ϕ29 capsid protein is located in a similar position as the equivalent in P22, and is found sitting above the E-loop of the underlying HK97-like fold.

**Figure 8:**
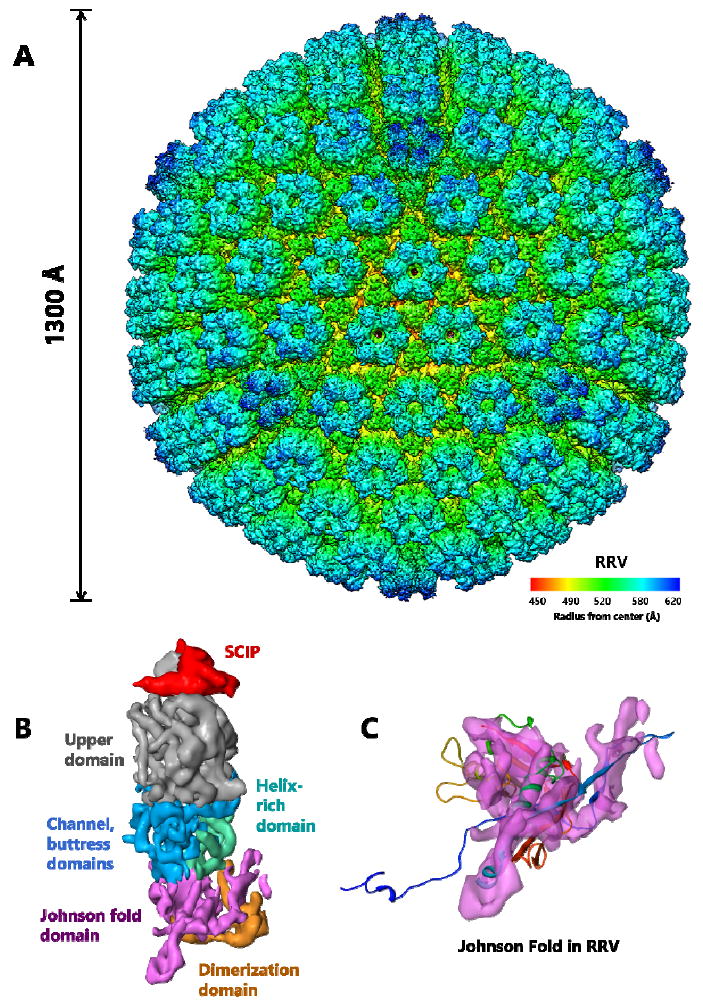
Chainmail in Herpesvirus RRV. (A) Even large, complex viruses such as the T = 16, 1300 Å herpesvirus RRV display the HK97-like fold. Side view of the major capsid subunit in herpesvirus RRV. (B) Each hexon subunit contains one monomer of SCIP (red) and one monomer of MCP. Each MCP monomer can be divided into six domains: upper domain in the upper region (gray), channel, buttress (blue) and helix-rich domains (green) in the middle region, and Johnson-fold (HK97-like fold) (purple) and dimerization domains (orange) in the floor region. (C) Fitting the HK97-like fold of HK97 (ribbon) into the Johnson-fold domain of RRV (semitransparent purple) highlights similarities between their HK97-like folds. Adapted from [7] with permission from the publisher.

It is conceivable that the additional domains surrounding the HK97-like fold in the herpesviruses are also insertions (Figure 8). Unlike phages, these viruses are of significant medical relevance with eight types known to infect humans. The eight known human herpesviruses are classified into three subfamilies, alpha, beta and gammaherpesviruses. Alphaherpesvirus and betaherpesvirus subfamilies include the well-studied herpes simplex virus type 1 (HSV-1) [23] and human cytomegalovirus (HCMV) [24], respectively. The two known human gammaherpesviruses, Kaposi’s sarcoma–associated herpesvirus (KSHV) and Epstein-Barr virus (EBV), are associated with lymphomas and other malignancies [25-27]. Sub-nanometer resolution capsid structures of these viruses have shown the presence of HK97-like fold in their MCP [7,10,28,29]. Each MCP monomer contains over 1300 amino acids and is organized into six domains: upper, channel, buttress, helix-rich, dimerization and HK97-like fold [7]. The T = 16 capsid shell is formed nearly entirely by the small (~280 amino acids) Johnson-fold domain of MCP through non-covalent chainmail. The other five domains of MCP are likely insertions to the Johnson-fold domain on both sides of the MCP floor. Whether these insertional domains are also attached to the HK97-like fold at the regions described above is yet to be established through high resolution studies.

## 7. Conclusion

Viruses likely originated from ancient cells through encapsulating cellular plasmids or genome fragments by cellular protein [30]. Indeed, cellular complexes resembling the structure and topology of HK97 gp5 have been found in both bacteria and archaea cells [3,4,31]. Similarly, it is natural to expect that cellular proteins with the BPP-1 topology of the HK97-like fold also exist elsewhere. The gene encoding one of these topologies might have evolved from the other through non-circular permutation in ancient cells through horizontal gene transfer—a rule for evolution in the prokaryotic world. In fact, although rare, non-circular permutation has been observed in cells, as exemplified by bacterial DNA methyltransferases [32-34]. Such permutation needs multiple steps of gene cutting-and-pasting [33], and can generate non-functional or less fit products. However, such non-functional intermediates may serve a purpose for cellular gene redundancy and lead to functional proteins through further evolution. These ancient cellular genes, encoding cellular proteins with the HK97-like fold, might have independently given rise to the viral proteins with different topologies and insertional domains. Adaptation to more complex environments of higher level organisms such as animal cells could have naturally led to acquisition of additional structures by way of insertions at locations of the loops of the HK97-like fold, as revealed in eukaryotic viruses such as herpesviruses. Such insertional domains could also give rise to auxiliary proteins by way of gene splicing such as the proposed case between BPP-1 and P22-like phages.

## Figures and Tables

**Table 1:** Comparison of Structures Based On Stabilization Strategy.

	Virus Name	Capsid Size	Triangulation Number	Johnson Fold	Basis of Chainmail	Chainmail Type	Resolving Method

**HK97 like**	**HK97**	660 Å	T=7	HK97-like	Isopeptide bonds	Covalent	X-ray Crystallography (3.44 Å)

**P-22 like**	**φ29**	450 Å wide	prolate T=3 Q=5	HK97-like	BIG2-like domain	Non-covalent	cryoEM (7.9 Å)
		540 Å wide					

	**P-22**	690 Å	T=7	HK97-like	I-domian	Non-covalent	capsid: cryoEM (4.0 Å), I-domain: NMR

	**Sf6**	690 Å	T=7	HK97-like	I-domian	Non-covalent	cryoEM (7.8 Å)

	**CUS-3**	690 Å	T=7	HK97-like	I-domian	Non-covalent	cryoEM (6.8 Å)

	**T4**	860 Å wide	prolate T_end_ = 13	HK97-like	I-domian	Non-covalent	gp24: X-ray Crystallography (2.9 Å), capsid: cryoEM (22 Å)
		1200 Å long	laevo T_mid_=20				

**BPP-1 like**	**BPP-1**	670 Å	T=7	BPP-1-like	Auxiliary Protein Dimer	Non-covalent	cryoEM (3. 5 Å)

	**ε15**	n/a	T=7	BPP-1-like	Auxiliary Protein Dimer	Non-covalent	cryoEM(4.5 Å)

**Lambda like**	**Phage λ**	600 Å	T=7	HK97-like	Auxiliary Protein Trimer	Non-covalent	gpd: X-ray Crystallography (1.1 Å), capsid: cryoEM (6.8 Å)

	**80α**	630 Å	T=7	HK97-like	Auxiliary Protein Trimer	Non-covalent	cryoEM (10.2 Å)

**More Complex**	**SIO-2**	800 Å	T=12	HK97-like	Auxiliary Proteins	Non-covalent	cryoEM(8.5 Å)

	**RRV (herpesvirus)**	1200 Å	T=16	HK97-like	Triplex hetero-trimers, dimerization domains,	Non-covalent	cryoEM (7.2 Å)

	**φRSL1**	1230 Å	T=27	HK97-like	Auxiliary Protein Trimer, Spike complex bridge between Trimers	Non-covalent	cryoEM (9 Å)
